# Inhibitory effect of STAT3 gene combined with CDDP on growth of human Wilms tumour SK-NEP-1 cells

**DOI:** 10.1042/BSR20160072

**Published:** 2016-06-03

**Authors:** Junrong Wang, Nina Zhang, Haijiang Qu, Guangxian You, Junhui Yuan, Caie Chen, Wenyi Li, Feng Pan

**Affiliations:** * Department of Laboratory Medicine, Wenling Maternal and Child Health Care Hospital, Wenling 317500, Zhejiang Province, China; † Department of Oncology, The Second People's Hospital of Wenling City (Cancer Hospital in Taizhou, Shanghai Tumor Hospital in Taizhou Branch), Wenling 317502, Zhejiang Province, China; ‡ Department of Pediatrics, Wenling Maternal and Child Health Care Hospital, Wenling 317500, Zhejiang Province, China; § Department of General Surgery, The First Affiliated Hospital of Soochow University, Suzhou 215006, Jiangsu Province, China

**Keywords:** BAX, cisplatin (CDDP), glucose regulatory protein 78 (GRP78), STAT3, Wilms tumour (WT)

## Abstract

To investigate the effects of signal transducer and activator of transcription 3 (STAT3) combined with cisplatin (CDDP) on the growth of human Wilms tumour (WT) SK-NEP-1 cell subcutaneous xenografts in nude mice and the possible mechanisms. Human WT SK-NEP-1 cells were subcutaneously transplanted to establish the BALB/c nude mice xenograft model. Mice were randomly divided into five groups: blank control group, adenovirus control group (NC group), STAT3 group, CDDP group and STAT3 plus CDDP group (combination group). Tumour volume and tumour weight were observed during the therapeutic process. The expression levels of STAT3, glucose regulatory protein 78 (GRP78) and BCL2-associated X protein (BAX) were evaluated by immunohistochemical analysis. Compared with the STAT3 group or CDDP group, the tumour weight and volume was significantly reduced in the combination group (*P*<0.05). No statistical significance was found in NC group compared with the blank control group (*P* > 0.05). Immunohistochemical analysis showed that STAT3, GRP78 and BAX protein levels in the combination group were significantly higher than those in STAT3 group and CDDP group (*P*<0.05). Exogenous STAT3 and CDDP may synergistically inhibit the xenograft tumour growth through up-regulation of BAX protein via GRP78.

## INTRODUCTION

Wilms tumour (WT), also known as nephroblastoma, is the most common type of paediatric malignant solid kidney tumour with an incidence rate of approximately 1–2 per 1000000 during childhood. Its peak incidence age was 3 years old and 80% was seen under 5 years of age [1]. Currently, the comprehensive treatment of surgery, chemotherapy and radiotherapy, has greatly improved the prognosis of patients with WT, with a 5-year disease-free survival rate increased to 75% to 85% [2]. However, because of lack of specific clinical symptoms, approximately 39.2% of patients had reached the clinical stage III or above at the time of diagnosis, [3]. Resistance to chemotherapeutic drugs is a main cause of the low efficacy with the therapy of stage III and IV disease or relapsed tumours. Therefore, novel intervention strategies to enhance the effectiveness of chemotherapeutic drugs and reduce their resistance are urgently in demand.

Signal transducer and activator of transcription 3 (STAT3) is a transcription factor that plays a key role in many cellular processes including cell growth and apoptosis. Studies have found that STAT3 gene has low expression in majority of tumour cells, suggesting that reduced STAT3 gene expression may be involved in tumorigenesis, [3]. STAT3 plays a pro-apoptotic role in many tumour cells, including kidney cancer, colon cancer, lung cancer and pancreatic cancer whereas up-regulation of STAT3 has no effect on normal cells [4–7]. Recent and previous studies have found that STAT3 protein can induce cancer cell apoptosis upon binding to the glucose regulatory protein 78 (GRP78), a chaperone located at the endoplasmic reticulum (ER) [8,9]. Another report has also showed that exogenous STAT3 combines with GRP78 and induces sensitization of H9c2 cardiomyocytes to hypoxia/reoxygenation (H/R), thus leading to cell apoptosis [10]. However, the function of STAT3 in Wilms tumorigenesis remains largely unknown.

Children with WT can be treated with actinomycin D and vincristine, the most classic and effective chemotherapy drugs pre- or post-surgery. Alternatively, cisplatin (CDDP) is commonly applied when this standard chemotherapy is ineffective. However, resistance to CDDP is still a major obstacle to the therapy. Strategies that enhance the effectiveness of CDDP are still required. Gene therapy, which targets tumour cells but has no effect on normal cells, becomes a promising strategy for cancer treatment [11]. A recent study has suggested that STAT3 may be involved in colorectal sensitization-fluorouracil [12]. In the present study, we investigated whether increased STAT3 expression could sensitize WT cells to CDDP. Our data showed that compared with the control groups, STAT3 combined with CDDP significantly suppressed WT cell growth in a xenograft model. Moreover, the mechanism by which STAT3 enhances the sensitivity of WT cells to CDDP may be due to increased BCL2-associated X protein (BAX) expression via GRP78.

## MATERIALS AND METHODS

The current study was approved by Research Ethics Committee of Wenling Maternal and Child Health Care Hospital.

### Materials

Human SK-NEP-1 cell line was purchased from CAS Shanghai Life Sciences Cell Resource Center, McCoy's 5A medium (Gibco), FBS, penicillin–streptomycin double-antibody (Hyclone), 30 female BALB/c nude mice with 4–6-week-old were purchased from Hayes Lake animal experiments limited liability company [licence number: SCXK (Shanghai) 2013-0004]. Matrigel (BD); STAT3 adenovirus and control adenovirus were purchased from Shanghai Genechem; CDDP, Nanjing pharmaceutical; STAT3 antibody, GRP78 antibody, BAX antibody (Cell Signaling Technology).

### SK-NEP-1 cell culture and WT xenograft tumour mouse model

SK-NEP-1 cell line was cultured in 15% FBS, 1% penicillin–streptomycin double antibody, McCoy's 5A medium under 5% CO_2_ at 37°C. Semi-adherent cells were grown in exponential growth phase, cells are directly slight pipetting off. After centrifugation at 125 ***g*** for 5 min, cells were resuspended in McCoy's 5A medium and Matrigel mixture and adjusted to a density of 1.5×10^7^/ml. With 1 ml sterile syringe vaccination, 0.2 ml SK-NEP-1 cell suspension was subcutaneously inoculated into the right front of nude mice. After injection, alcohol swab slightly and press against the cell fluid leakage at the inoculation site was required.

### Animal grouping and processing

After xenograft tumour growing up to 8–10 mm in diameter, mice were randomly divided into five groups, with six mice in each group: blank control group, adenovirus control group (NC group), STAT3 group, CDDP group and STAT3 plus CDDP group (combination group) respectively. Intratumoral injection of small amount, multi-point of 0.1 ml PBS, adenovirus (1.0×10^10^ pfu) or 1.5 g/l CDDP every second day for six times. Every third day, tumour volume was measured with a vernier caliper and calculated [*ab*^2^ × 0.5 (*a*: maximum tumour diameter, *b*: the shortest diameter of the tumour)]. Tumour inhibition rate was calculated as [1 − (treatment group mean tumour weight or volume/control group mean tumour weight or volume)] × 100%. Tumour growth curve was killed on day 22 and tumour blocks were stripped with sterile ophthalmic scissors and tweezers after weighing. For haematoxylin–eosin (HE) pathological and immunohistochemical analysis, tumour xenograft tissues were blocked with 4% paraformaldehyde for 24 h, and then treated with gradient alcohol dehydration (70% alcohol soaked 2 h, 80% alcohol soaked 3 h, 95% alcohol soaked 40 min, anhydrous ethanol for 30 min), transparent (xylene soaked 30 min), paraffin-embedded sections (thickness 4.5 μm) and HE staining pathological changes were observed in tumour tissues. By using immunohistochemistry streptavidin-perosidase (SP) method, the expression of STAT3, GRP78 and BAX was detected, and the mean absorbance at a wavelength of 570 nm of immunohistochemical staining was analysed by quantitative software ImagePro Plus6.0.

### Statistical methods

Data were analysed using the statistical software package (SPSS19.0). All data are expressed as mean ± S.D. (*x* ± S.D.). ImagePro Plus 6.0 software and GraphPad Prism 5 software were used for statistical analysis. Comparison between the two groups was analysed using *t* test, ANOVA and student-Newman-Keuls (SNK) method. *P* value <0.05 was considered to be statistically significant.

## RESULTS

### Tumorigenesis in nude mice

On the first day of nude mice inoculated with SK-NEP-1 cells, soya bean sample size vesicles were observed and then disappeared on the next day. At 18–20th day, a grain of rice-like tumour mass was observed. One week after establishment of subcutaneous xenografts in nude mice, the tumour mass grow significantly fast and substantially uniform in diameter up to 8–9 mm, suggesting that successful subcutaneous xenograft model in nude mice was established.

### Tumour volume was inhibited in combination group, STAT3 group and CDDP group

**Figure 1:**
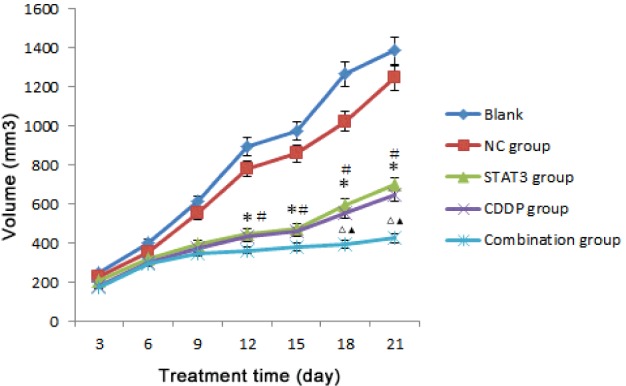
Tumour volume after different treatment time in each group. Compared with the blank control group *P<0.05; compared with the NC group, ^#^*P*<0.05; compared with CDDP group, ^△^*P*<0.05; compared with STAT3 group, ^▲^*P*<0.05.

Infection or necrosis was examined in the tumour inoculation sites. Tumour volumes in blank control group and NC group were significantly increased post-treatment compared with pre-treatment (1 328. 47±328. 76) mm^3^ compared with (249. 00±37. 01) mm^3^, (1 218. 08±307. 06) mm^3^ compared with (244. 75±37. 64) mm^3^ respectively. Although increased tumour volumes were found post-treatment in the blank control group and NC group, there was no significant difference (*P* > 0.05). Compared with the blank control group, the tumour volumes were significantly decreased in STAT3 group [624.21±54.21 compared with 1421.57±241.06 mm^3^], CDDP group [603.72±82.41 compared with 1421.57±241.06 mm^3^] and combination group [376.18±68.91 compared with 1421.57±241.06 mm^3^] respectively. Moreover, compared with STAT3 group and CDDP group, tumour volume in the combination group decreased more significantly (*P*<0.05). However, no significant difference was found in the tumour volume between STAT3 group and CDDP group (*P* > 0.05). Therefore, STAT3 or CDDP used alone have inhibitory effect on tumorigenesis and the combination of STAT3 and CDDP showed more significant inhibitory effect (Figure 1, *P*<0.05).

### Tumour weights and inhibitory rate in different groups

Xenograft tumours were irregular small round balls, with the top layer covered with pseudo fibrous capsule, and the surrounding tissue boundaries were very clear, and the cut surface showed fish-shaped with angiogenesis. Tumour weights in tumour-bearing mice were 0. 13±0. 02 g in STAT3 plus CDDP group, 0. 21±0. 06 g in STAT3 group, 0. 16±0. 04 g in CDDP group, 0. 37±0. 06 g in NC control group and 0. 38±0. 08 g in blank control group. No significant difference in tumour weights was found between NC control group and blank control group. However, STAT3 plus CDDP combination group showed increased inhibitory rate compared with that of STAT3 group or CDDP group, the differences were statistically significant (*P*<0.05, Table 1).

**Table 1:** Tumour weight and tumour inhibitory rate on intervention for day 22 in nude mice Compared with the blank control group, *P<0.05; compared with the NC control group, ^#^*P*<0.05; compared with CDDP group, ^△^*P*<0.05; compared with STAT3 group, ^▲^*P*<0.05.

Group	Tumour weight (g)	Tumour inhibitory rate (%)
Blank control group	0.38±0.08	–
NC control group	0.37±0.06	7.68
CDDP group	0.16±0.04^*#^	60.05^*#^
STAT3 group	0.21±0.06^*#^	48.68^*#^
Combination group	0.13±0.02^*#△▲^	67.84^*#△▲^

### Pathological changes in tumour blocks

**Figure 2:**
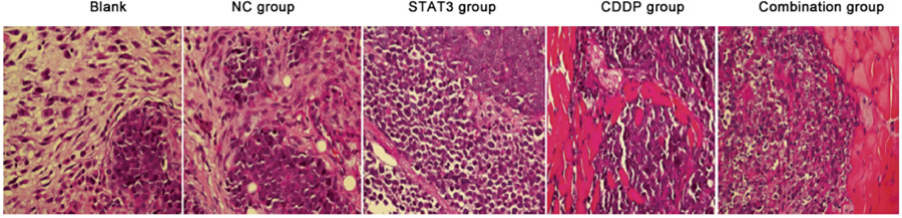
HE staining in subcutaneous xenografts in nude mice (×400) CDDP, cisplatin; NC group, adenovirus control group; STAT3, signal transducer and activator of transcription 3.

HE staining showed that in NC control group and blank control group, tumour necrosis area was very rare, and swarms of small round cells forming a sheet of diffuse distribution can be seen in primitive blastema tissue area with an increased nucleus cytoplasm ratio. After CDDP, STAT3 or combination treatment, cells were seen with common highly specific chromatin condensation, mitotic and apoptotic bodies (Figure 2).

### Immunohistochemical assay of associated proteins

**Figure 3:**
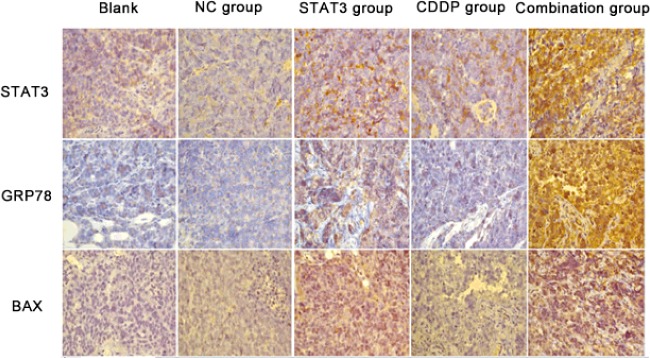
Expressions of STAT3, BAX and GRP78 in xenograft tumour tissues by immunohistochemical staining (×400) CDDP, cisplatin; NC group, adenovirus control group; STAT3, signal transducer and activator of transcription 3; BAX, BCL2-associated X protein; GRP78, glucose regulatory protein 78.

Immunohistochemical assay showed that in the combination group, expressions of STAT3, GRP78 and BAX were 0.26±0.04, 0.24±0.02, 0.23±0.01, which were significantly higher than those in the STAT3 or CDDP treatment groups, the differences were statistically significant (*P*<0.05). However, no statistically significant difference was found between CDDP group and STAT3 group [(0.12±0.02) compared with (0.11±0.02), *P* > 0.05, Figure 3 and Table 2].

**Table 2:** Quantitative expressions of STAT3, BAX and GRP78 of GRP78 in xenograft tumour tissues Compared with the control group, *P<0.05; compared with the control group adenovirus, ^#^*P*<0.05; compared with CDDP group, ^△^*P*<0.05; compared with STAT3 group, ^▲^*P*<0.05.

Group	Blank control group	NC control group	CDDP group	STAT3 group	Combination group
STAT3	0.13±0.01	0.14±0.01	0.16±0.01	0.17±0.01	0.26±0.04^△▲^
GRP78	0.11±0.02	0.13±0.01	0.12±0.02	0.14±0.02	0.24±0.02^△▲^
BAX	0.15±0.03	0.15±0.03	0.18±0.03	0.22±0.03	0.23±0.01^*#^

## DISCUSSION

Although 5-year survival rate has increased to above 90% in WT, drug resistance remains the main challenge for effective treatment of stage III and IV disease or relapsed tumours. CDDP is one of the commonly used chemotherapy drugs in solid tumours, but drug resistance limits its application. Hence, exploring strategies that could increase the sensitivity of cancer cells to CDDP is of significant importance. Since 1990s, gene therapy targeted to tumour cells with no effect on normal cells, has been a hot topic [11]. Combination of gene therapy and chemotherapy had been showed to increase the efficacy of therapy [12]. *In vivo* tumorigenicity experiment is the most intuitive and simple animal model which provides insight into the pathogenesis, diagnosis and treatment of WT [13]. Using the xenograft model, we found that overexpression of STAT3 significantly suppressed WT cell growth *in vivo*. In agreement with previous study [12], we also found that CDDP treatment effectively inhibited the growth of tumour-bearing mice tumour blocks. Moreover, combination of CDDP and STAT3 has more pronounced effect on tumour growth inhibition.

Previous literatures reported that STAT3 binds to the N-terminal domain of chaperone GRP78 and induces cell apoptosis [14,15]. GRP78, also known as the immune immunoglobulin heavy chain binding protein (BIP), is a heat shock protein 70 (HSP70) family member that mainly locates at the ER. GRP78 has showed to be highly expressed in tumour tissues, and involved in tumour cell invasion and migration. It has been showed that binding of STAT3 and GRP78 induce unfolded protein accumulation within the ER, leading to activation of unfolded protein response (UPR) that may induce apoptosis. Once the UPR signal was enhanced, GRP78 expression will correspondingly increase and binding to STAT3 to transport to the plasma membrane [16]. By binding to exogenous STAT3, GRP78 can also promote ER stress (ERS), which increases BAX expression on ER, leading to the ER damage, increases the outflow of calcium concentration in the cytoplasm and finally triggers the apoptosis [17]. In the present study, we found that both BAX and general regulatory factor 7 (GRF7) protein levels were significantly increased in the treatment of STAT3 combined with CDDP. Based on our results, we hypothesized that the combination of STAT3 and CDDP triggers UPR or ER by increasing GRP78 expression, which in turn up-regulated BAX protein levels, subsequently resulting in apoptosis of WT cells. However, further studies are still needed to figure out the detail mechanisms.

In summary, in the present study, we showed that combination of STAT3 and CDDP significantly suppressed growth of WT cells *in vivo*. The possible mechanism by which STAT3 enhances the sensitivity of WT cells to CDDP may be due to increased BAX expression via GRP78. These findings also suggest that combination of STAT3 and CDDP might be a potential strategy for WT therapy.

## Abbreviations

BAX: BCL2-associated X protein
CDDP: cisplatin
ER: endoplasmic reticulum
GRP78: glucose regulatory protein 78
HE: haematoxylin–eosin
NC group: adenovirus control group
pfu: plaque forming unit
STAT3: signal transducer and activator of transcription 3
UPR: unfolded protein response
WT: Wilms tumor
